# Bibliometric analysis of METTL3: Current perspectives, highlights, and trending topics

**DOI:** 10.1515/biol-2022-0586

**Published:** 2023-03-23

**Authors:** Hanqi Liu, Yanqing Huang, Shanshan Lu, Didi Yuan, Junwen Liu

**Affiliations:** Department of Histology and Embryology, Xiangya School of Medicine, Central South University, Changsha, Hunan, 420013, China

**Keywords:** METTL3, publications, bibliometrics, RNA methylation, epigenetics

## Abstract

N6-methyladenosine (m^6^A) is a representative of RNA methylation modification, which plays a critical role in the epigenetic modification process of regulating human diseases. As a key protein for m^6^A, methyltransferase 3 (METTL3) had been identified to be associated with a variety of diseases. The publications related to METTL3 were searched in the Web of Science Core Collection from the earliest mention to July 1st, 2022. Being screened by the retrieval strategy, a total of 1,738 articles related to METTL3 were retrieved. Much of our work focused on collecting the data of annual publication outputs, high-yielding countries/regions/authors, keywords, citations, and journals frequently published for qualitative and quantitative analysis. We found that diseases with high correlations to METTL3 not only included various known cancers but also obesity and atherosclerosis. In addition to m^6^A-related enzyme molecules, the most frequent key molecules were MYC proto-oncogene (C-MYC), Enhancer of zeste homolog 2 (EZH2), and Phosphatase and tensin homolog deleted on chromosome 10 (PTEN). METTL3 and methyltransferase 14 (METTL14) may function through opposite regulatory pathways in the same disease. “Leukemia,” “Liver Cancer,” and “Glioblastoma” were speculated to be potential hotspots in METTL3 related study. The number of publications had significantly surged year by year, demonstrating the growing importance of the research on epigenetic modification in the pathology of various diseases.

## 1 Introduction

**Figure 1:**
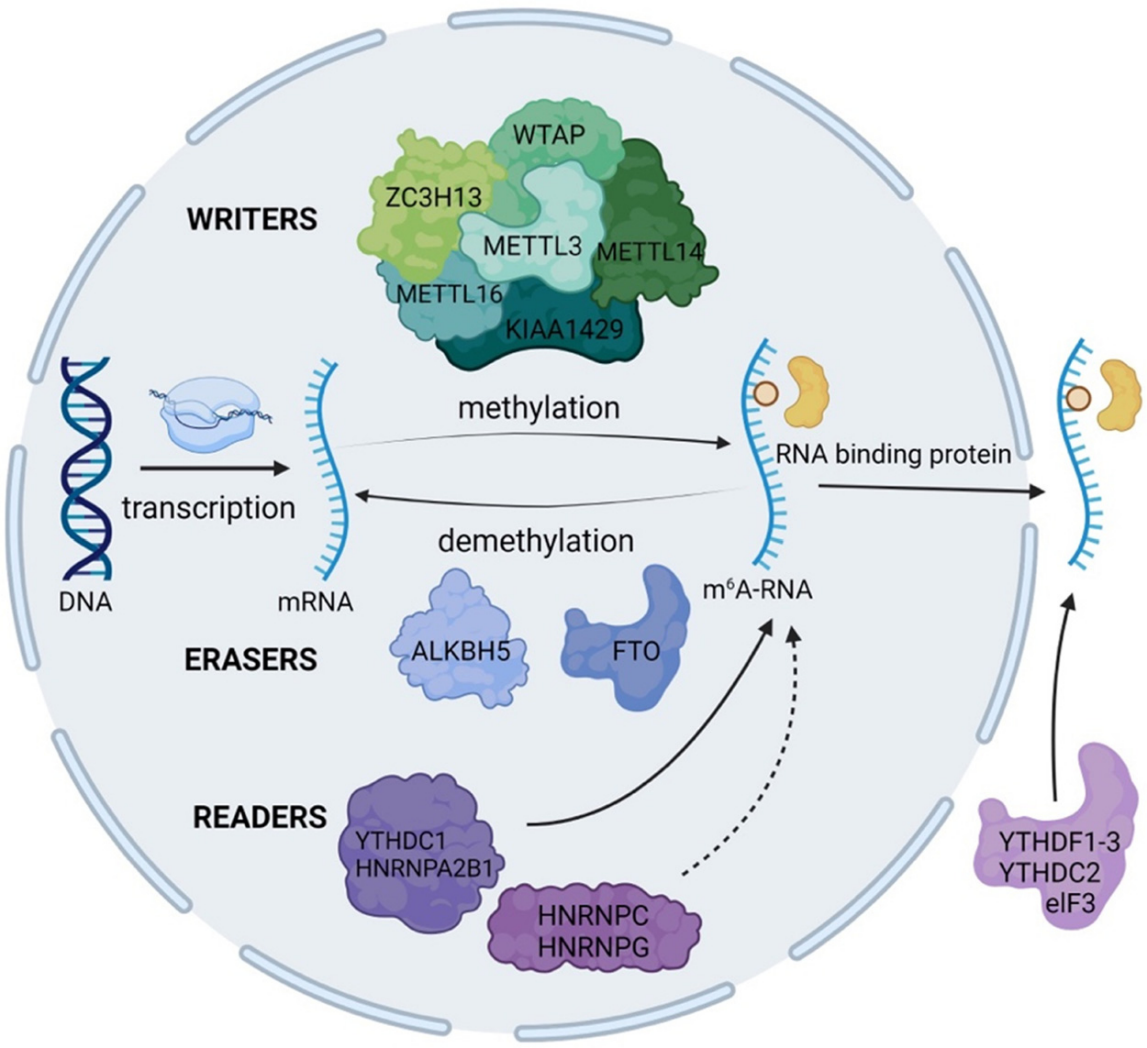
m^6^A methylation modification mechanism. Note: Writers, erasers, and readers are represented in different colors (green, blue, and purple, respectively). The structure of the membrane represented the nuclear membrane. For readers, the solid arrows indicate that the proteins directly interact with the substrate m^6^A-mRNA, and the dashed one indicate that the proteins did not directly bind to the m^6^A site, but mediate methylation modification by recognizing and binding to m^6^A-dependent structures.

As an emblematic type of RNA methylation, N6-methyladenosine (m^6^A) had been found to function by changing RNA structure or recruiting specific m^6^A binding proteins [1]. In 1994, Bokar et al. first purified a protein complex which can catalyze the formation of m^6^A, then scientists referred to the enzymes as m^6^A “writer” proteins [2]. Subsequently, the methylase system was gradually revealed, which consists of writers (catalyzation), erasers (removal), and readers (recognition). Summary of the m^6^A methylation modification mechanism is shown in Figure 1. These proteins regulate the methylation level of RNA in cells [3], and then affect the process of RNA nuclear export, degradation and translation. Writers perform indispensable functions in regulating the fate of mRNA in the first stage of methylation modification. Methyltransferase 3-methyltransferase 14 (METTL3-METTL14) is a vital heterodimer of multicomponent m^6^A methyltransferase complex (MTC), in which METTL3 constitutes the catalytic core [4].

METTL3 encodes the 70 kDa subunit of N6-adenosine-methyltransferase. In eukaryotic mRNAs, METTL3 plays a regulatory role through forming m^6^A to adjust the post-transcriptional methylation level of internal adenosine residues. Its related pathways include chromatin regulation/acetylation and processing of capped intron-containing pre-mRNA [2]. Existing studies show diseases associated with METTL3 mainly involved in oncology [5,6].

Bibliometric analysis is broadly used to visually calculate the academic impact of achievements in various disciplines [7]. Although it has several methodological limitations, it is still an effective tool for the assessment of scientific relevance in a specific field. It usually obtains and processes quantitative data from existing publications and then assesses the current research performance. Bibliometric analysis of publications can help to understand the research progress of METTL3, summarize and evaluate the current existing research results, as well as inspire researchers to explore more consequential treatment in the direction of epigenetics [8,9].

Although there had already been many publications on basic research in METTL3, we found that the bibliometric studies on METTL3 lack an overall and global analysis of the research progress and a systematic description. We tried to search for “METTL3 OR methyltransferase-like protein 3 OR methyltransferase like 3 protein” and “Bibliometric OR Bibliometrics” in the Web of Science Core Collection (WoSCC), but no relevant literature was retrieved. In view of the limitations of retrieval [10], there may exist some bibliometric publications on METTL3 not utilizing the words like “bibliometric(s)” in titles or abstracts, leading to their not being retrieved, but our research results still demonstrated that there were only few bibliometric studies on METTL3.

In this study, we had conducted our analysis on annual publication outputs, output contributors, journals, keywords, and citations to better assist researchers in selecting appropriate collaborators and research directions. The hotspots and frontiers of METTL3 were then mapped using VOSviewer, CiteSpace, and a bibliometric website. In this way, we expect our analysis to reveal the research pattern, highlight trending topics, and provide new inspiration for epigenetic researchers.

## 2 Materials and methods

### 2.1 Data source and search strategy

**Figure 2:**
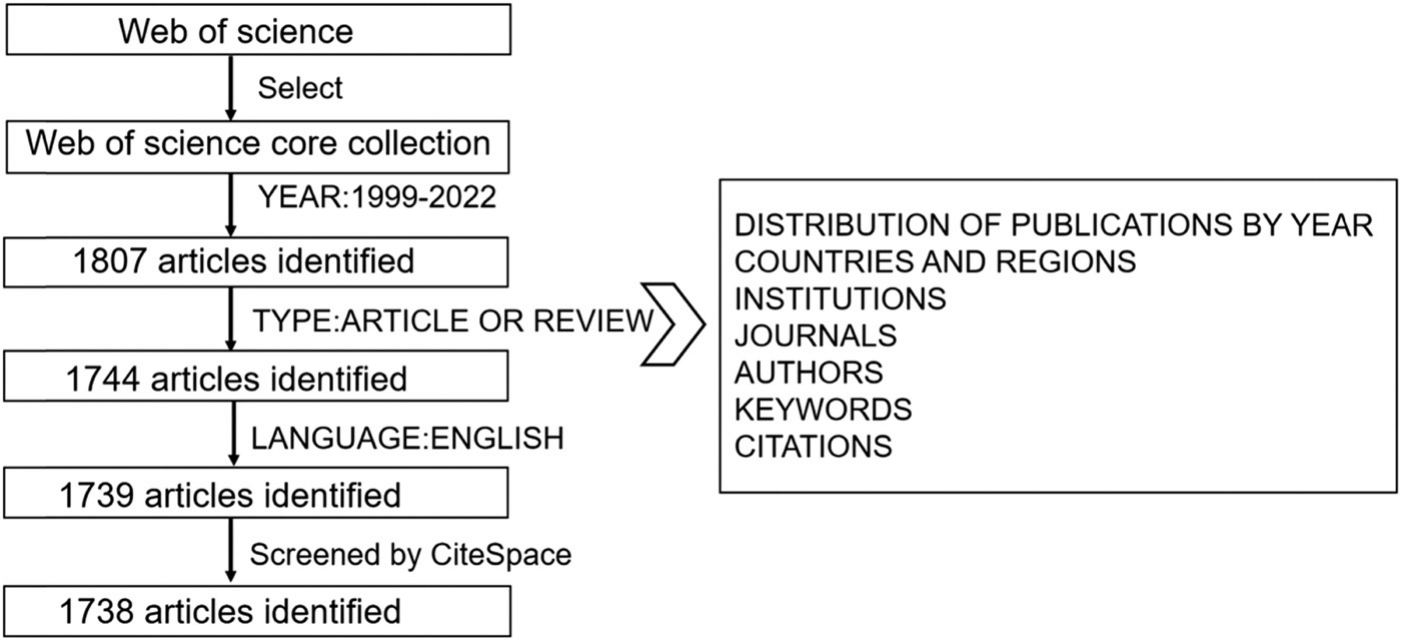
Flow diagram of search strategy in the screening of publications.

Relevant subject terms for METTL3 were first searched at NCBI (https://www.ncbi.nlm.nih.gov/mesh) to minimize misses. Then, literature retrieval was performed online through the WoSCC (https://www.webofscience.com) on July 1st, 2022. To avoid possible bias on account of daily database updation, we performed all searches within the same day. The research terms were as follows: TS = (METTL3 OR methyl-transferase-like protein 3 OR methyltransferase like 3 protein) refined by WEB OF SCIENCE INDEX (Web of Science Core Collection. SCI) AND DOCUMENT TYPES (ARTICLE OR REVIEW) AND LANGUAGES (ENGLISH), and the timespan was 1999–2022. The involved publications included the “early access.” We then utilized CiteSpace to remove duplicate literature from the retrieved results. The screening and analyzing process is illustrated in Figure 2.

### 2.2 Data collection

Raw data from the WoSCC were initially downloaded and verified. Then, the data were imported into Microsoft Excel, Bibliometric online analysis platform (https://bibliometric.com), VOSviewer (Version 1.6.18; https://www.vosviewer.com), and CiteSpace VI (Version 6.1.R2; https://CiteSpace.podia.com) and systematically analyzed.

### 2.3 Statistical methods

We performed analysis on publications included by publication years, countries, institutions, authors, journals, keywords, and citations, and tried to abstract their characteristics to get descriptive results. The figure of mechanism was created with BioRender.com. (www.biorender.com) CiteSpace VI was then utilized for constructing a correlation map in countries’ cooperation, and VOSviewer software for network visualizations in some cases such as institutions, co-authors, keywords, and citations. Besides, CiteSpace VI was also used to apply burst detection to investigate research directions and institutions with great research potential.

## 3 Results

### 3.1 Annual publication outputs

**Figure 3:**
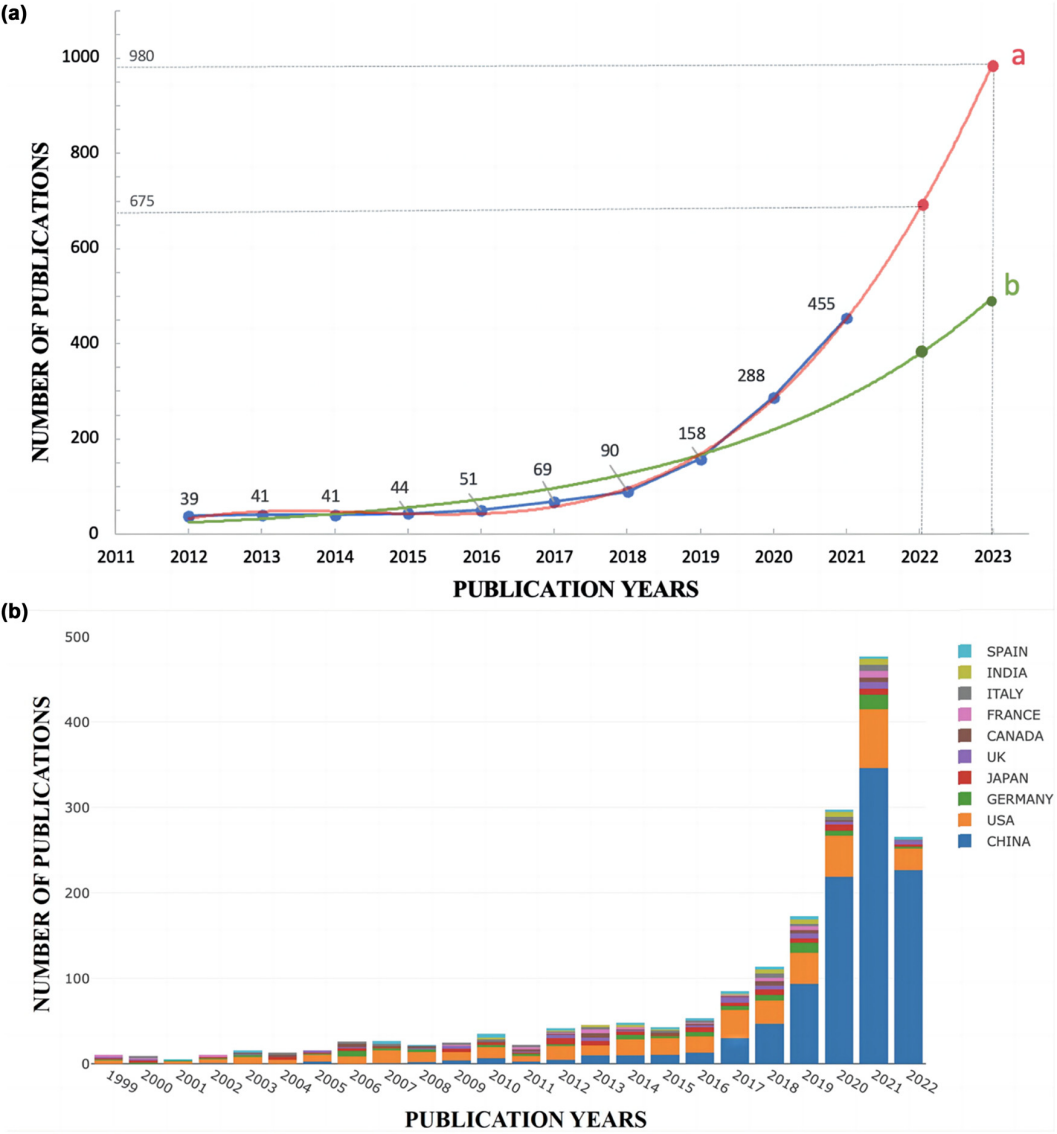
(a) Annual publication outputs of publications on METTL3. Note: The number of publications quickly surge year by year from 2012 to 2021, and since 2019, publications had an explosion of growth. Polynomial adjustment (a): *y* = 1.6078*x* ^3^ − 9716.8*x* ^2^ + 2 × 10^7^ *x* − 1 × 10^10^, *R* ^2^ = 0.9972. Exponential adjustment (b): *y* = 2 × 10^−237^e^0.2724^ *x*, *R* ^2^ = 0.9132). (b) The proportion of articles published by countries each year from 1999 to 2021. Note: Different colors represent publications number of different countries.

We counted the number of publications each year in the core journals of WoS. Overall, a total of 1,738 literature were included in our analysis. The number of publications in each year are shown in Table 1. Here the results illustrated that the number of publications from 1999 to 2011 was less than 30. However, since 2012, the publications began to surge, showing a sharp upward trend. To account for the law of publication trend, as shown in Figure 3a, exponential and polynomial fitting were both performed with the acquired data of annual outputs in the recent 10 years (2012–2021). The fitting equation of the exponential curve was *y* = 2 × 10^−237^e^0.2724^ *x* (with a correlation coefficient of 0.9132), and the polynomial curve fitting equation was *y* = 1.6078*x* ^3^ − 9716.8*x* ^2^ + 2 × 10^07^ *x* − 1 × 10^10^ (with a correlation coefficient of 0.9972).

**Table 1:** Number of publications in each year

Year	Documents	Year	Documents	Year	Documents	Year	Documents
1999	10	2005	16	2011	21	2017	69
2000	8	2006	21	2012	39	2018	90
2001	7	2007	27	2013	41	2019	158
2002	12	2008	18	2014	41	2020	288
2003	16	2009	21	2015	44	2021	455
2004	16	2010	30	2016	51	2022*	239

^*^Result statistics as of July 1st, 2022.

It could be observed that since 2019, the number of METTL3 publications had an explosion of growth, and the number of literature in 2020 was nearly twice that in 2019. Polynomial curve fitting was more consistent with METTL3 publication trend. By July 1st, 2022, the number of literature had reached 239 in 2022. Given that the publications were consistent with the results of polynomial fitting, total number of publications in 2022 was expected to reach 675 and 980 in 2023. In aggregate, the publication number had increased rapidly in the past 10 years, which suggested METTL3 had become a research hotspot year by year, showing self-evident importance.

The top 10 countries by the number of publications each year are shown in Figure 3b in different colors, which presents the evidence that China and USA contributed the most to the total publications. From 2011, China began to emerge, and the number of publications showed a steady upward trend. After 2019, publications number in China steadily accounted for more than half of the total number. The rapid growth of publications in China was the main reason for the noticeable increase in the total number of literature, which indicated that compared with other countries all over the world, China may have a larger population base for scientific research in this field. According to the bar chart, USA had begun to study on METTL3 since 1999, and the number of publications in the past 2 years had also increased.

### 3.2 Countries and regions

**Figure 4:**
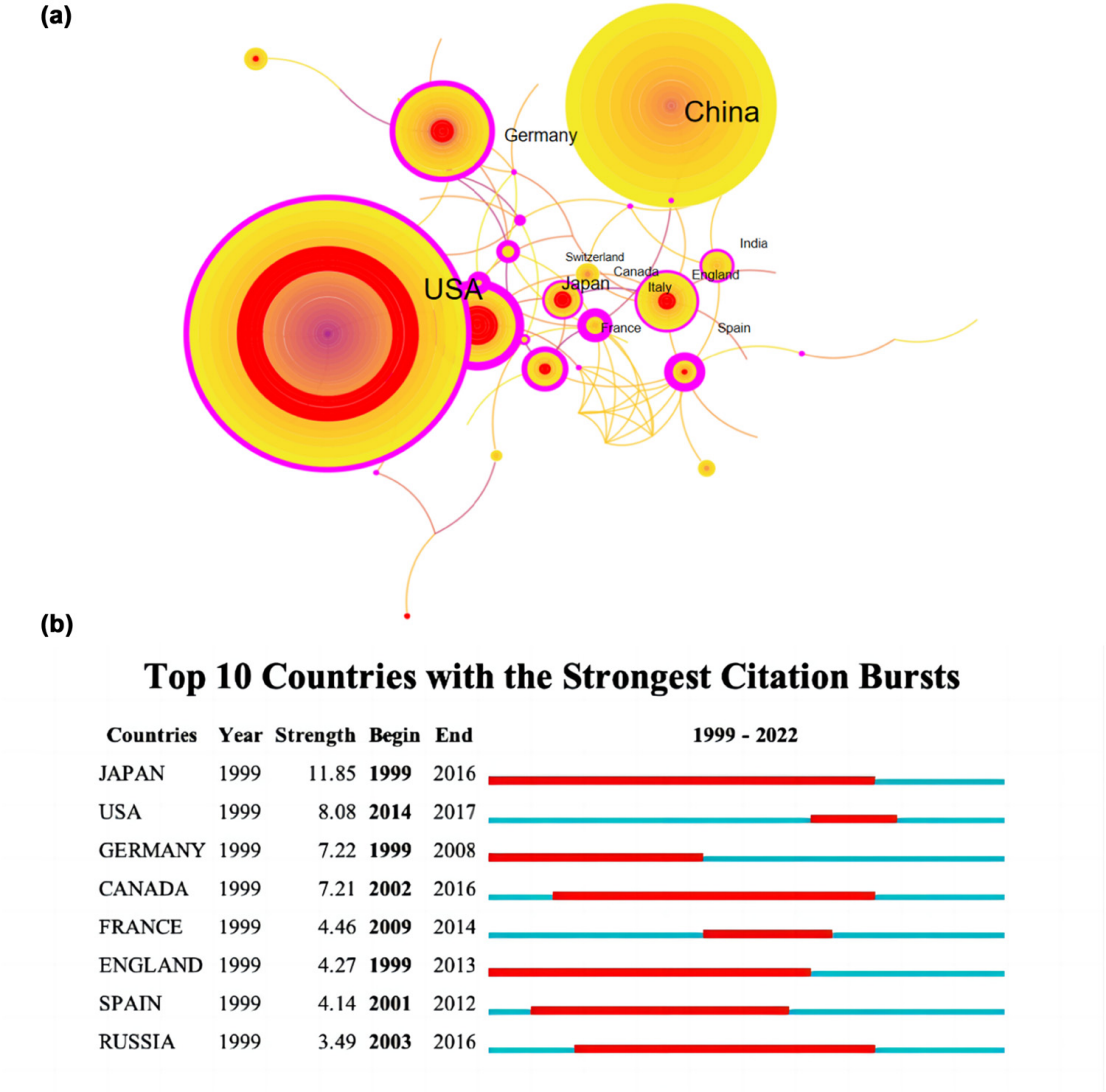
(a) Analysis of cooperation among the top 10 countries/regions with the highest documents. Note: The size of each node presents its number of documents, and the proportion of the outermost ring means its centrality. Central red circles illustrate nodes’ temporal importance. (b) Top ten countries/regions with the strongest citation bursts. Note: The strongest citation burst means that a variable changes greatly in a short period. Red bars illustrate the duration of the burst.

The 1,738 publications on WoSCC were contributed by 58 countries/regions. The top 10 countries by citation counts are shown in Table 2. Our major evaluation indicators were citation counts, centrality, total link strength, bursts, and sigma. Sigma is constructed by combining centrality with bursts, which represents the nodes’ novelty. The top ranked item by citation counts were China (*n* = 1,023, 46.8%), pursued by USA (*n* = 424, 19.4%), and Germany (*n* = 88, 4.0%). Extensive collaboration between countries/regions are shown in Figure 4a. The purple circles around nodes represent centrality indicating the pivotal role in cooperative relationships among countries/regions. Node with high mediating centrality is often a key hub connecting two different domains. The country with the highest centrality is Spain (0.44), followed by Japan (0.43) and Italy (0.42). Citation counts of China was almost half the number; however, its centrality was very low. While only a few documents were published by Spain and Italy, the two countries were still important hubs in the network of study on METTL3.

**Table 2:** Top 10 countries by citation counts

RANK	Country	Occurrence	Centrality	Burst	Sigma
1	China	1,023	0	0	1
2	USA	424	0.26	8.08	6.61
3	Germany	88	0.21	7.22	4.06
4	Japan	79	0.43	11.85	70.35
5	England	53	0.18	4.27	2.02
6	Canada	46	0.14	7.21	2.62
7	France	37	0.28	4.46	2.98
8	Italy	36	0.42	0	1
9	India	35	0.11	0	1
10	Spain	29	0.44	4.14	4.53

We also analyzed the bursts of each country’s publications, which showed in the form of central red circles in Figure 4a. Bursts indicate the temporal importance of nodes. Detailed information of top 10 countries with strongest citation bursts are shown in Figure 4b. Japan (11.85), USA (8.08), and Germany (7.22) are in the top three. Among them, Japan had the largest burst length, which lasted from 1999 to 2016, and was in the first group of countries that began study on METTL3. Thus, combined with the above data, Japan indeed had strong centrality in the publications network. Remarkably, the shortest burst length that lasted for only 3 years from 2014 to 2017 belongs to USA, but its publications were the second prolific, which elucidated that METTL3 related research in USA was rapid and concentrated.

### 3.3 Institutions

Over 1,703 institutions had contributed to the publications on WoSCC. The institutions, documents, citations, and centrality are shown in Table 3. In relation to institutions with the highest number of publications, Sun Yat-Sen University (93), Zhejiang University (74), and Chinese Academy of Sciences (69) are in the top three. Among them, document citations of Chinese Academy of Sciences far exceeded other institutions, reaching 6,588. The top 10 institutions with publications number are distributed in China (*n* = 9) and USA (*n* = 1). Although the publications were only 38, the University of Chicago’s citations were far higher than any other institution, with 7,195.

**Table 3:** Institutions ranked by documents

Rank	Institutions	Country	Documents	Citations	Centrality
1	Sun Yat-Sen University	China	93	2,987	0.03
2	Zhejiang University	China	74	4,962	0.04
3	Chinese Academy of Sciences	China	69	6,588	0.12
4	Nanjing Medical University	China	63	2,507	0.03
5	Shanghai Jiao Tong University	China	60	2,027	0.02
6	Central South University	China	45	777	0.01
7	University of Chinese Academy of Sciences	China	45	3,896	0.05
8	Fudan University	China	40	1,887	0.03
9	The University of Chicago	USA	38	7,195	0.07
10	Zhengzhou University	China	35	908	0.02

**Figure 5:**
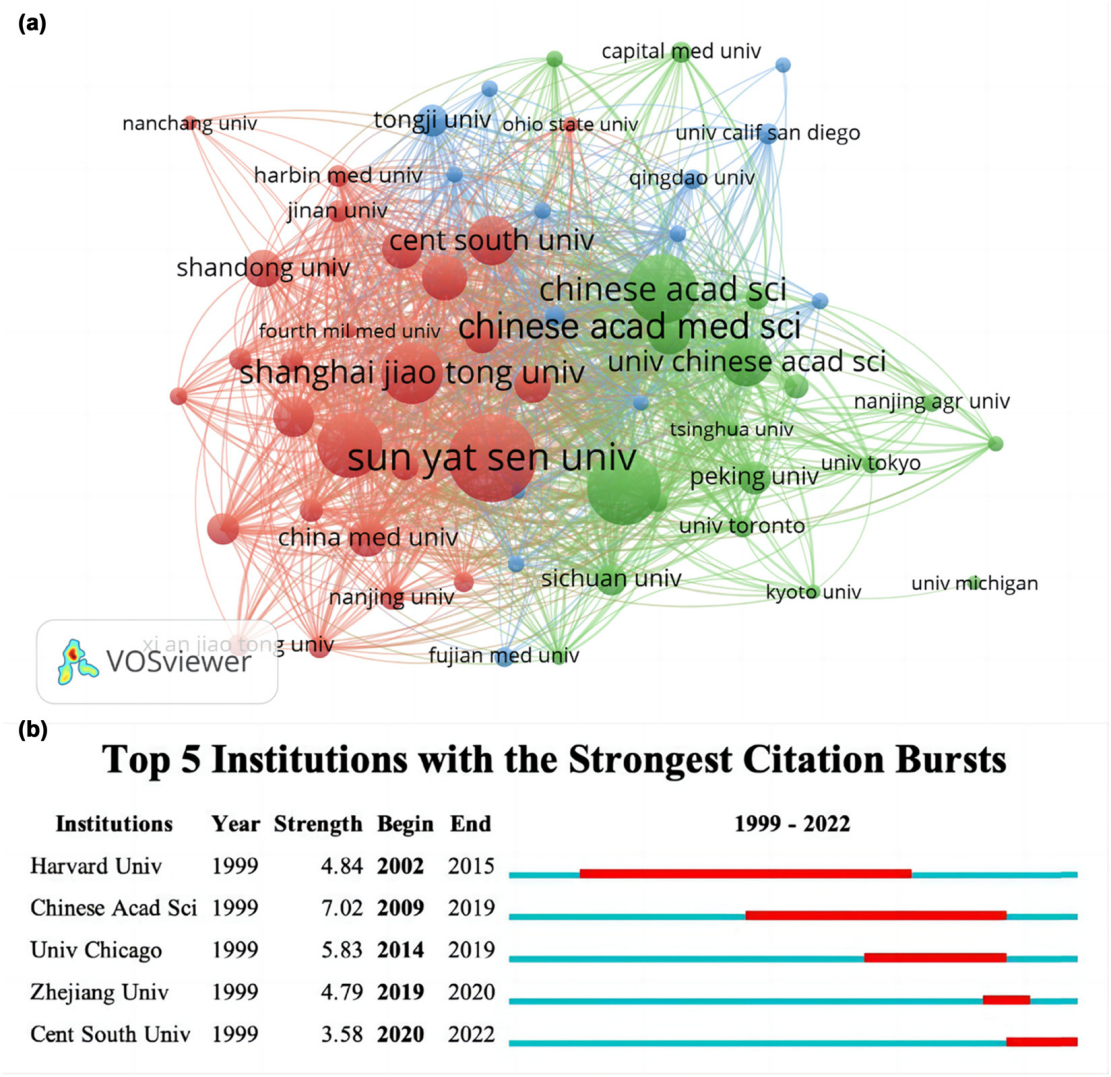
(a) Visualization graphs of correlation between institutions. Note: The main institutions were classified into three clusters, presented by three colors (red, blue, and green). Node size indicates number of publications by institutions. Lines between nodes showed cooperative relationships among organizations, and thicker lines between two nodes indicate stronger link strength. (b) Top five institutions with the strongest citation bursts. Note: The strongest citation burst means that a variable changes greatly in a short period. Red bars illustrate the duration of the burst.

We then utilized VOSviewer to construct a relationship network between institutions, which reflect the cooperative relationships between organizations. The institutions are classified into three groups in Figure 5a, represented by three colors (red, blue, and green). Node size indicates the number of articles published by institutions. Lines between nodes represent the cooperative relationships among organizations, and the line thickness showed the link strength between two nodes and evaluated the degree of cooperation among organizations. Chinese Academy of Sciences had the highest centrality (0.12), and it can also be observed in Figure 5a that it played a pivotal role in the central position. Comparing the centrality of countries in Table 2 with that of institutions in Table 3, it showed that the centrality of country is generally higher, which indicated that cooperation between institutions was less.

Top five institutions with strongest citation bursts are shown in Figure 5b, among which Central South University has shown high enthusiasm in the research during the past 2 years (2020–2022). The outbreak of Harvard University lasted for 13 years (2002–2015), indicating that in the early stage of METTL3 research, Harvard University showed a strong research interest and may have had a deep foundation now.

### 3.4 Co-author

**Figure 6:**
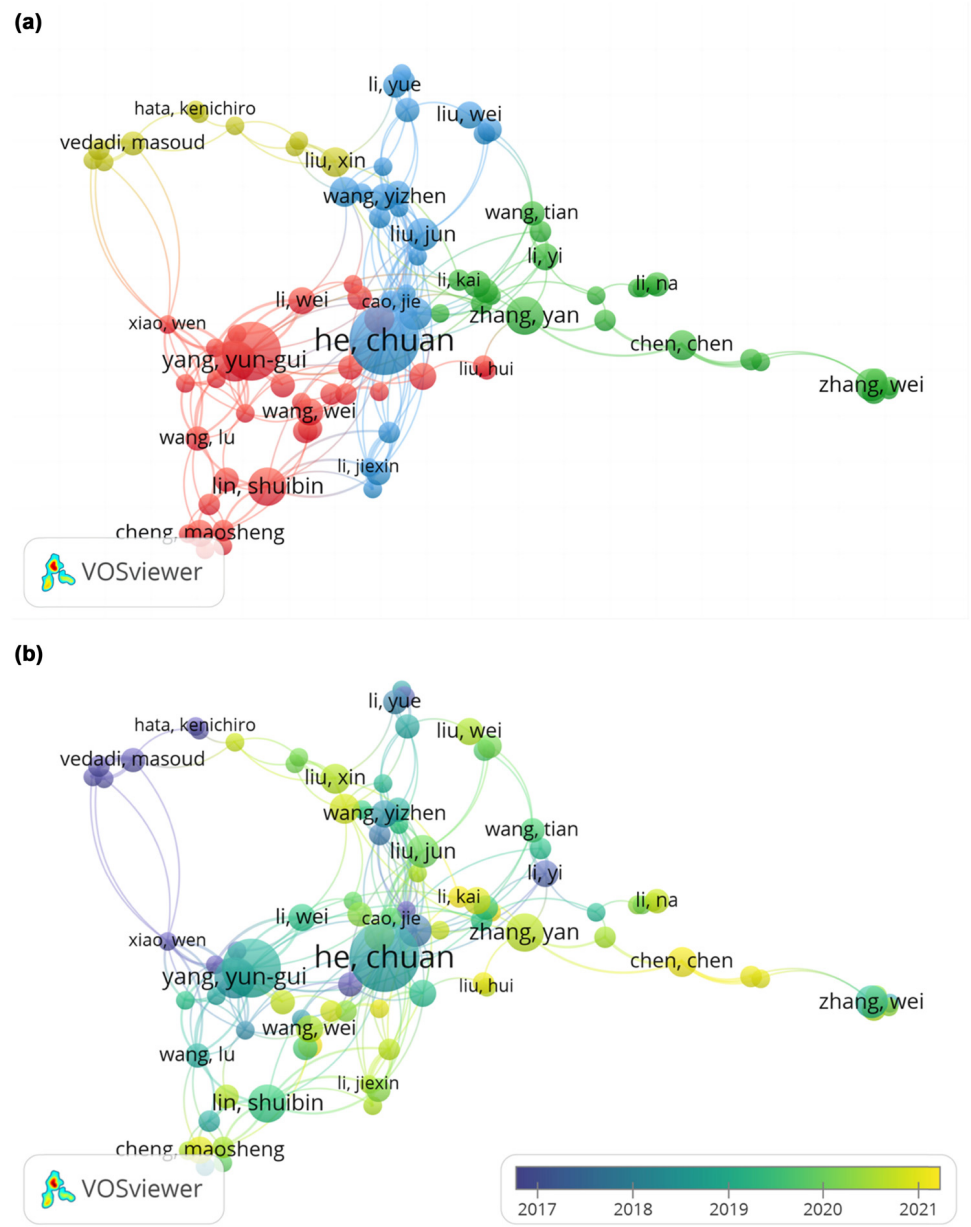
Visualization graphs of co-authorship analysis of authors. (a) Network visualization of authors involved in studies on METTL3. Note: Total number authors were classified into four clusters with different colors (red, blue, green, and yellow) to show different major cooperative groups. Node size indicates publications number. Lines between nodes showed cooperative relationships among organizations, and thicker lines between two nodes indicate stronger link strength. (b) Overlay visualization of authors involved in studies on METTL3. Note: Different colors showed different period of publications for each node.

More than 10,999 authors contributed to the writing of 7,038 documents. Taking 5 as the minimum number of the documents of an author, 104 authors meet the threshold. A total of 91 authors were used to construct the author correlation network, excluding authors who were not directly related to other authors. The correlation diagram in Figure 6a consists of 91 items, 262 links, and 4 clusters (red, blue, green, and yellow), and there were 2 main cooperative groups. The core of blue group was He Chuan’s team, which also served as the core in the whole research network and linked main cooperative groups. The cores in red were Yang Ying’s team and Lin Shuibin’s team. Remarkably, there was no direct connection between the two teams, which shed light on that the two teams may have studied relatively independent important topics. Yang Ying’s team mainly focused on the methyltransferase centered with METTL3. Among their research results, “Mammalian WT1 Associated Protein (WTAP) is a regulatory subunit of the RNA m^6^A methyltransferase [11]” has been cited up to 1,285 times, which illustrated that the RNA-binding capacity of METTL3 is strongly reduced in the absence of WTAP, revealing key factors that may influence METTL3 function. However, Lin Shuibin’s team concentrated more on how METTL3-mediated m^6^A RNA methylation regulates bone marrow mesenchymal stem cells’ fate and osteoporosis directions [12]. Further exploration on these topics may have potential research prospects. As to the time network diagram in Figure 6b, we found essential publications mainly concentrated in 2019. Plenty of autonomous groups had started to emerge in the last 2 years, indicating that more investigators were joining the METTL3 research cohort.

Then, we further analyzed the detailed information of the top ten authors by citations. The main authors and their affiliations, Hirsch index (H-index), documents, citations, and total link strength are shown in Table 4. Among these indices, H-index helps to understand the importance and impact of a researcher’s cumulative research contribution. Total link strength indicates how many links are connected to that node (including the number of repetitions), demonstrating the level of correlation between researchers of that node and other. According to affiliations, most of the authors belong to China (*n* = 7), and the rest were from USA (*n* = 1) and Norway (*n* = 1). Among these authors, He Chuan (116), Liu Jianzhao (54), and Yang Yungui [39] had the top three H-index, playing important roles in the network, and this result was also consistent with data of total link strength.

**Table 4:** Pivotal authors of documents

RANK	Author	Affiliation	Country	H-index	Documents	Citations	Total link strength
1	He, Chuan	University of Chicago	USA	116	25	4,700	44
2	Lu, Zhike	Westlake University	China	47	7	3,294	18
3	Liu, Jianzhao	Zhejiang University	China	54	10	2,904	36
4	Yang, Ying	Wuhan University	China	16	20	2,675	43
5	Yue, Yanan	Zhejiang University	China	16	5	2,235	19
6	Yang, Yungui	China Natl Ctr Bioinformation	China	39	13	2,202	36
7	Wang, Yang	Hospital of Harbin Medical University	China	20	6	1,823	7
8	Wang, Xiao	Nankai University	China	8	8	1,786	7
9	Lin, Shuibin	Sun Yat-Sen University	China	22	12	1,637	20
10	Zhao, Xu	National Hospital Norway	Norway	7	5	1,506	15

### 3.5 Journals

METTL3 related publications had been published in 617 journals from 1999 to 2022 (by July 1st). Information of the top ten journals by number of publications are listed in Table 5. Impact factor (IF) was utilized to evaluate the significance of a journal (https://clarivate.com/webofsciencegroup/essays/impact-factor/). According to the Journal Citation Reports (JCR) 2021 standards, IF of journals in Table 5 range from 3.75 to 69.50, with an average of 18.39. Among the top ten journals, Nature had the highest IF of 66.85 in 2021, followed by Molecular Cancer (41.44) and Nucleic Acids Research (19.16), which showed more authority in this field and indicated that METTL3 had high research value and prospects. The top three journals in which the articles were published were Journal of Biological Chemistry (40, 2.30%), Frontiers in Cell and Developmental Biology (40, 2.30%), and Frontiers in Oncology (35, 2.01%), respectively. As such, it was more likely to find the valuable references we need in these mentioned journals.

**Table 5:** Top ten journals with the largest number of documents

Rank	Journal	2021 IF	Count	% of 1,738
1	Journal of Biological Chemistry	5.49	40	2.30
2	Frontiers in Cell and Developmental Biology	6.08	40	2.30
3	Frontiers in Oncology	5.74	35	2.01
4	Nucleic Acids Research	19.16	27	1.55
5	Cell Death & Disease	9.71	26	1.50
6	Molecular Cancer	41.44	25	1.44
7	Nature Communications	17.69	24	1.38
8	Nature	69.50	22	1.27
9	Journal of Cellular and Molecular Medicine	5.30	22	1.27
10	PLOS ONE	3.75	22	1.27

### 3.6 Keywords

**Figure 7:**
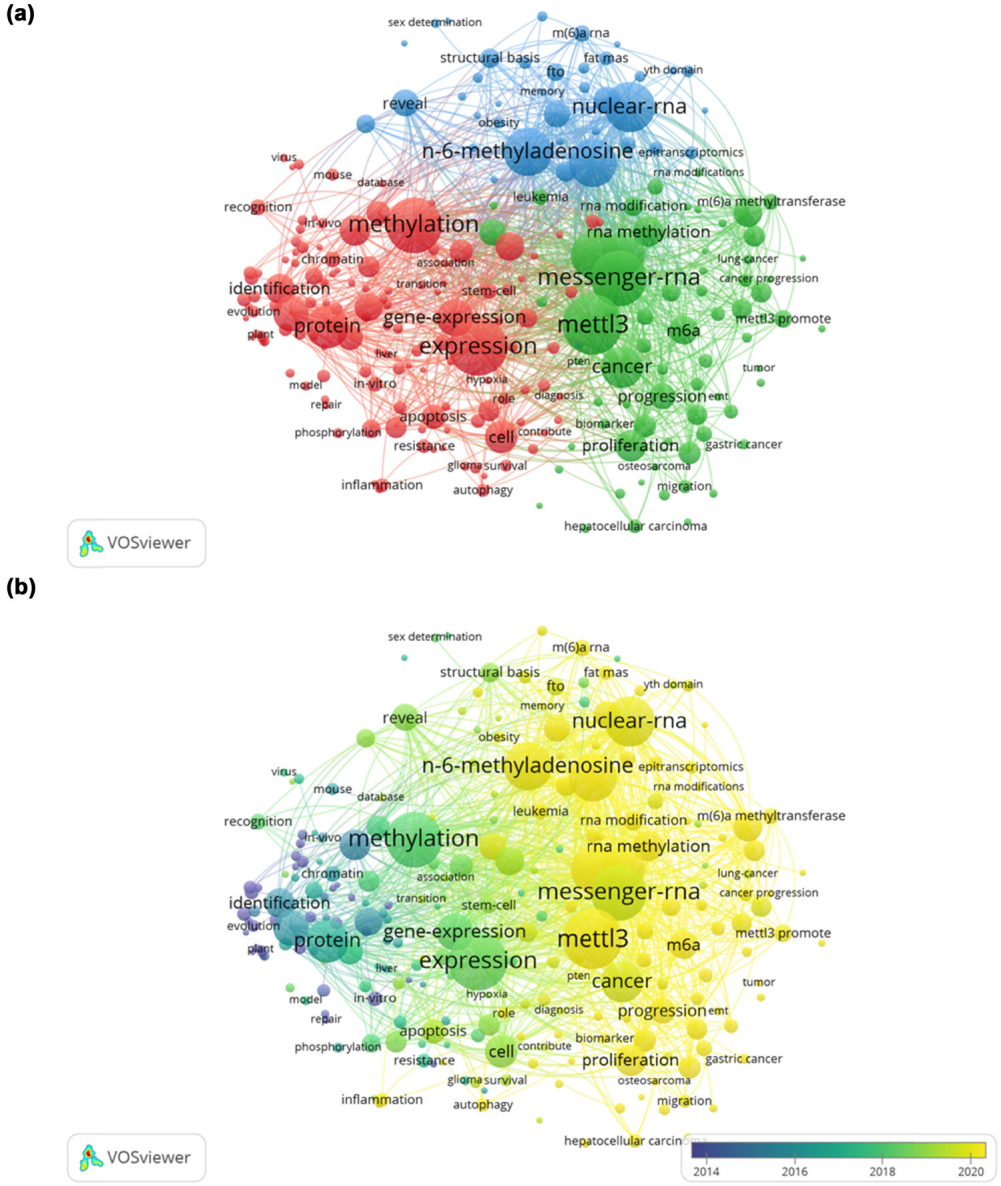
Visualization graphs of keywords. (a) Network visualization of keywords in METTL3. Note: Total number of authors were classified into 3 clusters with different colors (red, blue, and green) to show different research theme. Node size shows the frequency of keywords. Lines between nodes showed cooperative relationships among organizations, and thicker lines between two nodes indicate stronger link strength). (b) Overlay visualization of keywords in METTL3. Note: Different colors showed different periods of publications for each node.

To present the knowledge map of keywords for METTL3 research, a total of 7,089 keywords were extracted from titles and abstracts. Taking 10 as the minimum number of occurrences of a keyword, of the 7,089 keywords, 237 met the threshold. VOSviewer was utilized to construct a relationship network between keywords, which can reflect hot topics visually and show how research hotspots change over time. Theme words were classified into three clusters, as shown in Figure 7a represented by three colors (red, blue, and green). Depending on the keywords network was clustered by years, as shown in Figure 7b, the research focus had gradually shifted from cell-level research like “Stem cell” to disease research such as “fat mass” and “cancer” over time. Table 6 shows the top ten diseases, key molecules, and states among the filtered keywords, which indicated mainstream topics and frontiers in METTL3 related research.

**Table 6:** Top ten diseases, key molecules, and states in studies on METTL3

Rank	Disease	Occurrence	Molecule	Occurrence	State	Occurrence
1	Hepatocellular carcinoma	90	FTO	72	Proliferation	154
2	Breast cancer	85	ALKBH5	69	Differentiation	107
3	Colorectal cancer	57	METTL14	35	Progression	90
4	Leukemia	38	C-MYC	21	Apoptosis	81
5	Prostate cancer	25	WTAP	21	Metastasis	63
6	Glioblastoma	22	Histone H3	19	EMT	45
7	Obesity	22	EZH2	17	Invasion	39
8	Gastric cancer	19	YTHDF1	15	Inflammation	34
9	Lung cancer	18	YTHDF2	15	Tumorigenesis	33
10	Atherosclerosis	14	PTEN	10	Autophagy	30

It can be found that the METTL3 related studies were mainly concentrated in cancer. The top three diseases were “Hepatocellular Carcinoma,” “Breast Cancer,” and “Colorectal Cancer.” Therefore, we could put more effort on in-depth research of these areas. The molecules most closely related to METTL3 were “Fat Mass and Obesity Associated (FTO),” “AlkB Homolog 5 (ALKBH5),” and “METTL14.” Similarly, the top three states were “Proliferation,” “Differentiation,” and “Progression.”

**Figure 8:**
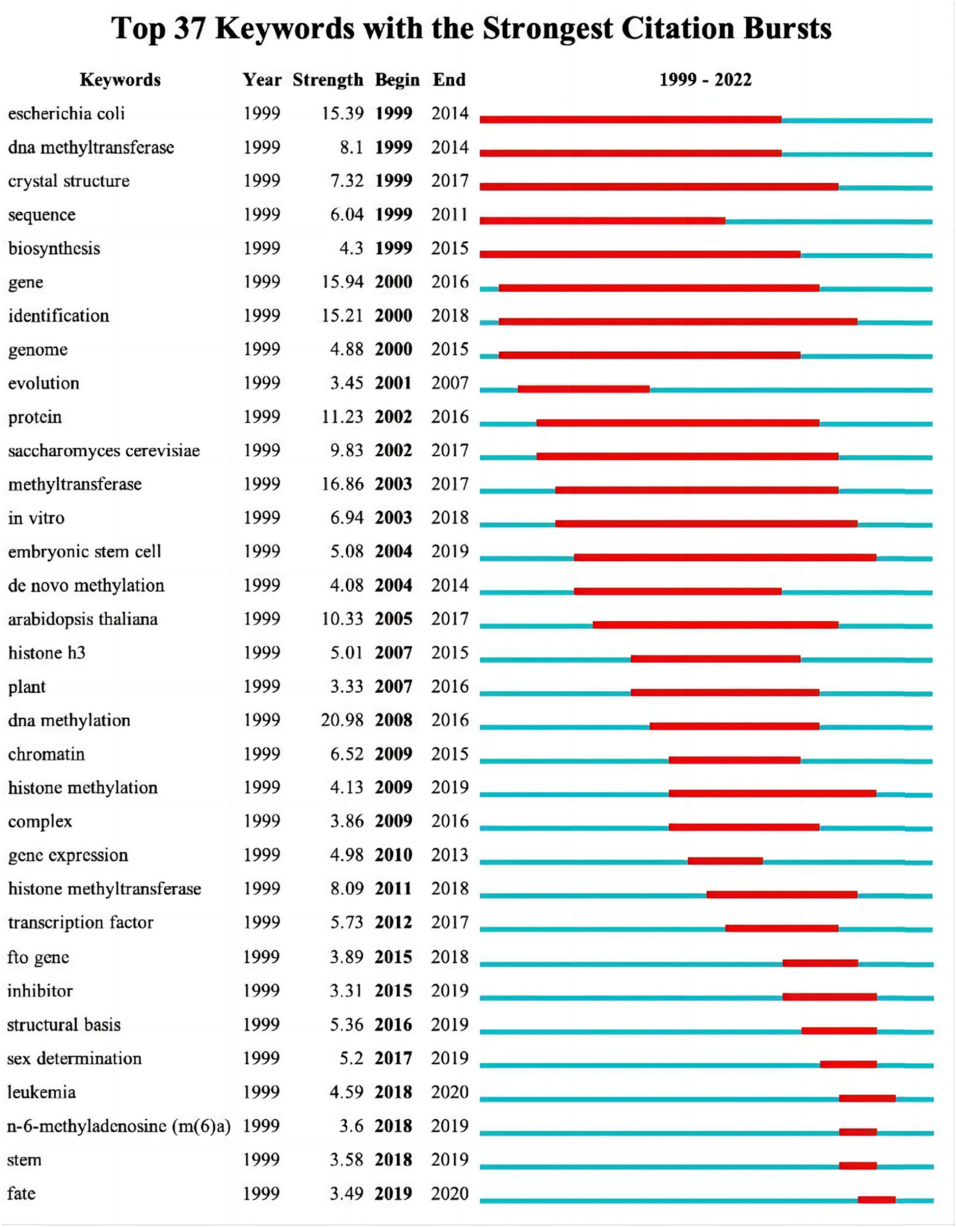
Top 37 keywords with the strongest citation bursts. Note: The strongest citation burst means that a variable changes greatly in a short period. Red bars illustrate the duration of the burst.

CiteSpace was utilized to detect burst keywords, which represented words that had been cited frequently over a period and indicated research frontier topics. By analyzing extracted keywords, we found 37 entries that suggested outbreak trends, as shown in Figure 8. Among them, DNA methylation (2008–2016), Histone methylation (histone methyltransferase) (2011–2018), and m^6^A (2018–2019) articulated that study on METTL3 shifted from DNA to histones, and the current research focus is m^6^A methylation on RNA. Arabidopsis Thaliana (2005–2017, 10.33), Histone H3 (2007–2015, 5.01), and FTO (2015–2018, 3.89) appeared to be an increasing trend in corresponding time periods. Research on Embryonic Stem Cells (2004–2019) had lasted for 15 years, and now more attention is devoted to leukemia (2018–2020). In addition, research from 2017 to 2019 suggested that METTL3 may also be involved in sex determination.

### 3.7 Citations

Table 7 shows the details of top ten articles with highest citations including title, journal, first Author, year, citations, and links. Higher the value of link, more nodes are connected with others (excluding the number of duplicates), indicating the correlation between publications. Themes of highly cited literature were mainly concentrated in the fields of stem cells, cancer cells, and germ cells, illustrating MTTTL3 plays a part mainly in the processes of cell division, differentiation, and transcription. Ranked first, “Auxin: Regulation, action, and interaction [13]” was published by Annals of Botany in 2005 and was cited 1,434 times in total. The second and third articles in turn were “A METTL3-METTL14 complex mediates mammalian nuclear RNA N6-adenosine methylation [4]” and “Mammalian WTAP is a regulatory subunit of the RNA m^6^A methyltransferase [11],” both had been published in 2014. Throughout the journals published literature with high citations, “Nature” and “Cell research” appeared more frequently, indicating METTL3-related articles published in these journals were more favored by scholars.

**Table 7:** Top ten highly cited publications related to METTL3

Rank	Title	Journal	First author	Year	Citations	Links
1	Auxin: Regulation, action, and interaction.	Annals of Botany	Woodward, AW	2005	1,434	1
2	A METTL3-METTL14 complex mediates mammalian nuclear RNA N6-adenosine methylation	Nature Chemical Biology	Liu, JZ	2014	1,384	186
3	Mammalian WTAP is a regulatory subunit of the RNA N-6-methyladenosine methyltransferase	Cell Research	Ping, XL	2014	1,026	153
4	Extensive translation of circular RNAs driven by N-6-methyladenosine	Cell Research	Yang, Y	2017	886	20
5	m(6)A mRNA methylation facilitates resolution of naive pluripotency toward differentiation	Science	Geula, S	2015	859	135
6	Meiotic catastrophe and retrotransposon reactivation in male germ cells lacking Dnmt3L	Nature	Bourc’his, D	2004	805	8
7	The m(6)A methyltransferase METTL3 promotes translation in human cancer cells	Molecular Cell	Lin, SB	2016	757	148
8	N-6-methyladenosine modification destabilizes developmental regulators in embryonic stem cells	Nature Cell Biology	Wang, Y	2014	741	115
9	m(6)A RNA methylation promotes XIST-mediated transcriptional repression	Nature	Patil, DP	2016	722	78
10	Perturbation of m6A writers reveals two distinct classes of mRNA methylation at internal and 5′ sites	Cell Reports	Schwartz, S	2014	660	103

**Figure 9:**
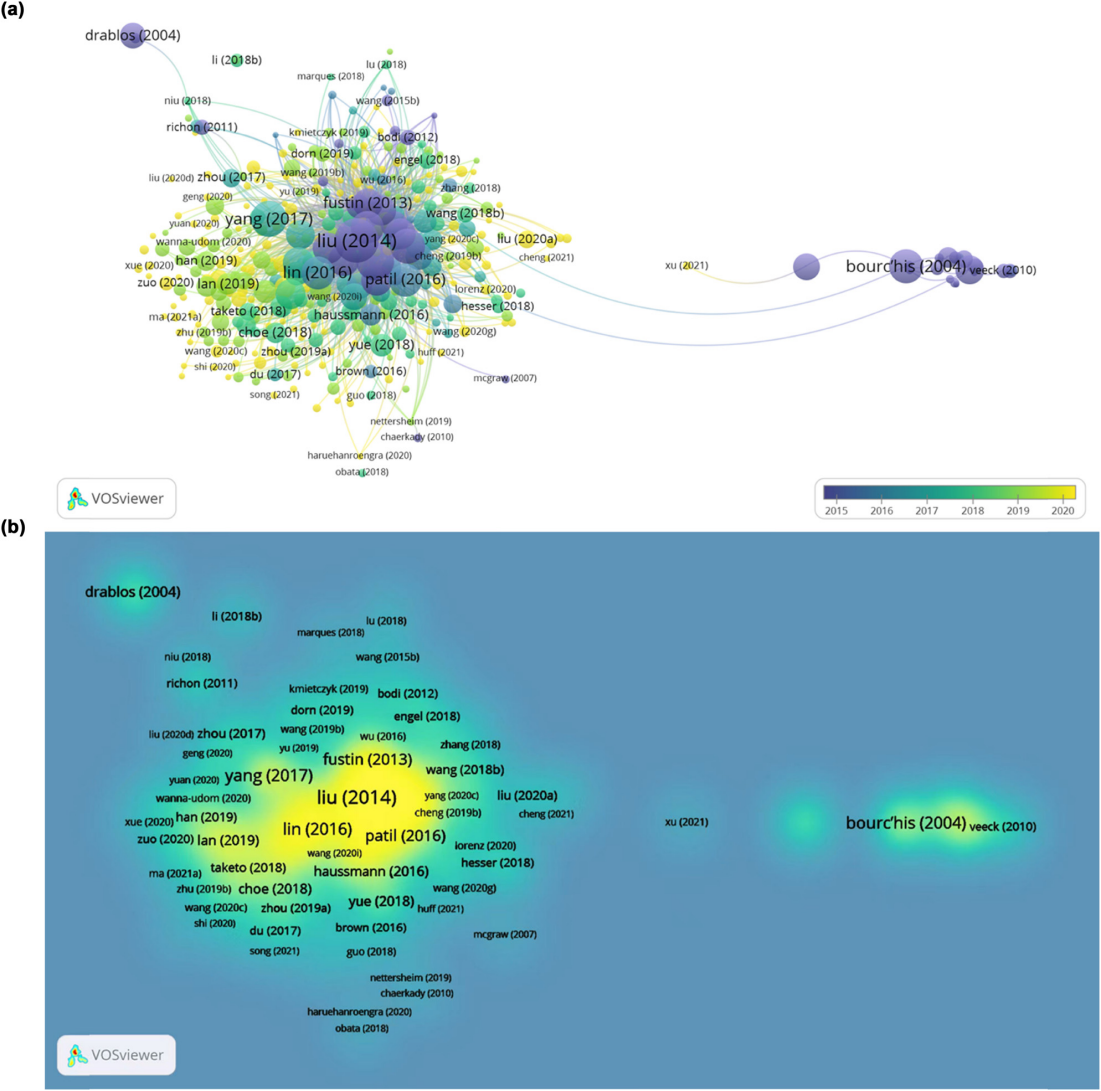
Visualization graphs of citations analysis. (a) Overlay visualization of citations involved in studies on METTL3. Note: Different colors show different periods of publications for each node. (b) Further, in Figure 9b, each item represents a literature, and the more frequently a literature is cited, the color of the point closer to yellow. On the contrary, the less frequently the literature is cited, the color of the point closer to blue.

We also constructed temporal and density networks of cited articles with VOSviewer, data on this topic over the past years are shown in Figure 9a. Further, in Figure 9b, each item represents a literature, and the more frequently a literature is cited, the color of the point closer to yellow. On the contrary, the less frequently the literature is cited, the color of the point closer to blue. It can be observed that the article published by Liu et al. in 2014 occupies a central position in the correlation network. In the first half of 2022, their article had been cited more than 1,384 times and it had a high link strength of 186 with other literature, linking the individual publications. The METTL3 research network was heavily influenced by articles that came out around 2014, indicating additional fresh discoveries were urgently needed to strengthen and expand the research network.

Co-citation can reveal the potential topics of a research field by analyzing the clusters of highly cited topics. As shown in Table 8, we statistically analyzed title, journal, first author, years, and frequency. The top five co-citations were all cancer-related, including leukemia (*n* = 2), liver cancer (*n* = 1), and glioblastoma (*n* = 1), indicating that these could all be potential topics with research prospects. In the existing research, there are few studies of METTL3 related to the diseases like glioblastoma. The article entitled “m^6^A RNA Methylation Regulates the Self-Renewal and Tumorigenesis of Glioblastoma Stem Cells [14]” published by Cui et al. in the Cell Reports has been repeatedly cited as a reference by METTL3 related articles, indicating that the research results of this article have played an important role in explaining how METTL3 functions in diseases. The role of METTL3 in glioblastoma could be a new research direction, and it is more likely to find new research results for scientists.

**Table 8:** Top five highly co-citation of cited references on METTL3

Rank	Title	Journal	First author	Year	Freq.
1	The m(6)A methyltransferase METTL3 promotes translation in human cancer cells	Molecular Cell	Lin, SB	2016	316
2	RNA N6-methyladenosine methyltransferase-like 3 promotes liver cancer progression through YTHDF2-dependent posttranscriptional silencing of SOCS2	Hepatology	Chen, MN	2018	282
3	The N-6-methyladenosine (m(6)A)-forming enzyme METTL3 controls myeloid differentiation of normal hematopoietic and leukemia cells	Nature Medicine	Vu, LP	2017	254
4	m(6)A RNA methylation regulates the self-renewal and tumorigenesis of glioblastoma Stem Cells	Cell Reports	Cui, Q	2017	229
5	Promoter-bound METTL3 maintains myeloid leukemia by m(6)A-dependent translation control	Nature	Barbieri, I	2017	213

## 4 Discussion

Bibliometric analysis is progressively being utilized as an essential tool for analyzing existing publications and assessing the trends in various research fields. m^6^A methylation modification plays a critical role in the research of epigenetic modification and attracts attention of more and more scientific research communities. As a leading molecule in the study of m^6^A methylation modification study, METTL3 had been put into great interests and research enthusiasm by lots of scientists. However, to date, there has been no bibliometric analysis of METTL3-related studies. This study used bibliometric methods to comprehensively analyze the current perspectives and trending topics of METTL3 research from 1999 to 2022 (up to July 1st, 2022). Based on inclusion and exclusion criteria, we adjusted the original results of 1,807 METTL3-related papers published in WoSCC, and finally screened 1,738 articles for inclusion in our analysis.

In 2012, methylated RNA immunoprecipitation sequencing (MeRIP-seq), an m^6^A antibody enrichment technique combined with high-throughput sequencing, first revealed the m^6^A modification profile in the entire human transcriptome [15]. This reversible eukaryotic post-transcriptional regulation opens up new areas for studying physiological and pathological processes, and also provides a technical tool for scientists’ research [16]. Likewise in this year, the number of METTL3-related publications have steadily increased. According to our fitting analysis of publications in the past 10 years from 2012 to 2021, starting from 2019, publications number had even far exceeded the exponential growth and entered a period of rapid surge. In this case, based on our polynomial fitting results, the total number of publications in 2022 is expected to reach 675 and 980 in 2023. China and USA occupied main position in the number of publications. Publications in each country increased significantly in the last 2 years, illustrating that the research on METTL3 had entered an active phase worldwide. It is well-known that there are many diseases regulated by METTL3, but its detailed molecular mechanism and how to target METTL3 to regulate m^6^A modification related diseases need more intensive research. The increase in the number of literature indicated that it was more likely to obtain new findings from the existing huge research data to promote the research process.

As to publications output contributors in global landscapes, China had a large number of publications but with a low centrality. On the contrary, even though a few articles had been published by Spain and Italy, the centrality of which were still high. Japan was one of the first group of countries that started to study METTL3, and the research in USA was rapid and centralized. Analyzing from the institutional level, Chinese institutions were firmly in the top of the world in terms of publications number, but the University of Chicago had high citations, which may be largely related to He Chuan’s team affiliated with the University of Chicago. The number of publications and the institutions devoted to research in China were relatively high, but articles published by western countries had a greater impact in this field. In addition, out of 10,999 authors, He Chuan’s team was at the core of the research network, with a high number of publications and H-index, and his team was also a pioneer team in epigenetic research. The paper “A METTL3-METTL14 complex mediates mammalian nuclear RNA N6-adenosine methylation [4],” with He Chuan as the corresponding author, proposed that METTL14 and WTAP act together as a complex of METTL3, which was a breakthrough milestone in METTL3-related research and had an important impact in the epigenetic field.

Further, keywords analysis indicated that METTL3 played a significant role in oncology. “Hepatocellular carcinoma” appeared as the most frequent keyword, followed by “Breast cancer” and “Colorectal cancer.” Overexpression of METTL3 was pertinent with tumorigenicity [17]. Research showed that METTL3 substantially overexpressed in HCC patients and has been identified as an adverse prognostic factor [18]. As to hepatocellular carcinoma, Pan et al. explicitly narrated the METTL3-related mechanism in HCC, which revealed the potential research prospects of METTL3. METTL3 targets Suppressor of Cytokine Signaling 2 (SOCS2), RAD52 Motif-containing 1 (RDM1), SNAI1, Hypoxia-Inducible Factor 1α (HIF-1α), LINC00958 and Forkhead box O3 (FOXO3) in HCC, and has a significant impact on the occurrence and development of the disease [19]. Therefore, further exploration on METTL3 combined with the abovementioned downstream target RNA may be an important potential research direction in HCC. In addition, as the most lethal cancer in women worldwide, 2.1 million females globally are presently afflicted by breast cancer, the epigenetic mechanism of which has received more and more attention [20]. The positive feedback loop of HBXIP/let-7g/METTL3/HBXIP was proposed to accelerate proliferation of breast cancer cells and then aggravate the process of HCC [21]. BCL-2 was also the target of METTL3, which regulated breast cancer’s proliferation and apoptosis in a m^6^A modification pathway [22]. The latest study related to colorectal cancer published in 2023 showed that METTL3 influenced the proliferation, migration, and invasion of colorectal cancer cells through regulating Notch1 and Hsa_circ_0000390 [23]. From our results, “FTO” and “ALKBH5” usually work together with METTL3 to regulate disease progression through epigenetic pathways. Above analysis elucidates the critical role of epigenetic mechanisms in oncology. In the latest literature published in 2023, breast cancer [24] and colorectal cancer [25] accounted for a significant proportion of publications, illustrating that these diseases were still the main research areas where METTL3 plays a regulatory role. By combining the proven upstream and downstream molecules of METTL3 in these diseases, we can further explore the role of METTL3 or directly research on how METTL3 affected the occurrence and development of these diseases through m6A modification pathways, which were both extremely promising research directions.

Apart from tumor-related diseases, “Obesity” and “Atherosclerosis” also appeared frequently in our results, suggesting that METTL3 may influence multiple pathways in other diseases. As a highly conserved single-standard and non-coding small RNAs with a length of 19–24nt, miRNAs play significant roles in development, differentiation, and fat deposition [26]. 67% of 3′-UTR regions containing m^6^A sites had at least one miRNA binding site [26]. On the one hand, as a major part of methyltransferase, METTL3 regulated the methylation level on mRNAs and affected the binding of miRNAs to target genes. On the other hand, METTL3 promoted the transformation of pri-miRNA into mature miRNA. It can also regulate adipocyte differentiation by directly modifying key genes [27]. The abovementioned ways allowed METTL3 influence adipocyte fate in a variety of ways. Some other studies revealed that METTL3-centered MTC positively controlled adipogenesis by promoting cell cycle transition during adipogenesis [28]. Therefore, it is reasonable to speculate that METTL3 may have a substantial regulatory function in diseases related to lipid metabolism. It is of great significance to explore METTL3’s role in lipid metabolism related diseases such as “obesity” and “atherosclerosis.”

Intriguingly, a recent study conducted a multi-omics analysis of METTL3 and METTL14 in HCC, illustrating that the two enzymes influenced stability or translation efficiency of mRNAs in an m^6^A dependent manner, then jointly regulate multiple signaling pathways and biological processes [29]. As core components of MTC, METTL3 and METTL14 are crucial in the process of methylation modification. In some other independent studies on the same cancer, METTL3 and METTL14 also have an opposite effect on tumor process [17,30]. We had found that “leukemia” showed an outbreak trend from 2018 to 2020, which is also why the keyword appears frequently, indicating a potential research direction. Martin and Park published “Follow-up with METTLs in Normal and Leukemia Stem Cells” in 2018 [31], which summarized three independent studies that identified METTL3 and METTL14 as vital regulators of differentiation in acute myelocytic leukemia pathogenesis, elucidating the important regulatory role of METTL3 in the pathogenesis of leukemia and further exploration may help to reveal the epigenetic mechanism in the pathogenesis of leukemia. Additionally, simultaneous research on METTL4 may further reveal opposite epigenetic pathways regulating the disease.

In addition to m^6^A-related enzyme molecules, the most frequent key molecules were C-MYC, EZH2, and PTEN. C-MYC is a kind of immortalized gene, which makes cells to proliferate indefinitely. As a master transcriptional factor, it regulates approximately 10–15% of genes in the genome and has been identified to be closely related to tumorigenesis [32]. METTL3 along with C-MYC regulatory pathways have been verified in oral squamous cell carcinoma [33], lung cancer [34], bladder cancer [35], etc. EZH2 encodes a histone lysine N-methyltransferase. This gene has a strong relationship with DNA methylation, and inhibit transcription of other genes [36]. It had already been verified that it was connected to nasopharyngeal carcinoma [37], breast cancer [38], and lung cancer [39], which were all identified to be regulated by epigenetic pathways via the METTL3-EZH2 pathway. A well-known tumor suppressor gene PTEN can inhibit tumor growth by antagonizing phosphorylases like tyrosine kinase from activating [40]. According to existing studies, diabetic nephropathy [41], diabetic retinopathy [42], ovarian cancer [43], hypoxic pulmonary hypertension [44], prostate enlargement [45], and some other diseases were associated with METTL3-PTEN pathway. Deeper study on these molecules can be more likely to quickly find diseases regulated by METTL3 and reveal their molecular pathways in pathogenesis of the specific disease.

Co-citations analysis present the evidence that “Leukemia,” “Liver Cancer,” and “Glioblastoma” were the major themes in the high frequency co-cited literature. Therefore, we reasonably speculate that research focused on these diseases would become potential hotspots in METTL3 related study. Focusing in these areas might more likely provide some valuable new discoveries. Among these diseases, there are few studies on the direct regulation of METTL3 on the occurrence and development of glioblastoma. The existing studies suggest that METTL3 can promote the development of glioblastoma stem cells in the m^6^A modification pathway. As mentioned above, the highly cited literature expounded overexpression of METTL3 gene suppresses glioblastoma stem cells growth and self-renewal [14]. Shi et al. recently focused on the role of METTL3 in glioma drug resistance [46]. Tassinari et al. demonstrated that METTL3 upregulation increases ADAR1 mRNA methylation modification and its protein level leading to a pro-tumorigenic [47]. Targeting the promoting effect of METTL3 on glioblastoma or further exploring the influence of downstream molecules such as ADAR1 on the disease perhaps could open up a new field of epigenetics in glioblastoma.

Though we had followed certain bibliographic principles and comprehensive analysis strategies, it is inevitable to lose some articles. During our retrieval, some articles may not have been involved because of not having keywords in their title or abstract. In addition, for the reason that some recently published high-quality articles had not appeared for a long time, the citation frequency cannot accurately evaluate their scientific value. Despite such limitations, given the lengthy publication retrieval timespan and sufficient number of publications, our analysis enables us to build a comprehensive picture of research trends and provide an instructive perspective for METTL3 research. Researchers should also pay more attention to the latest publications and keep up with the frontier research, which is more likely to discover potential significant research directions.

## 5 Conclusion

Rapid growth trend of publications on METTL3 proves its great research prospects. As far as we were concerned, this article has published the first bibliometric analysis of trends in METTL3 research. Our results show that China, USA and Germany were the top three countries contributing to METTL3 studies. Active cooperation was observed between developed countries. Sun Yat-Sen university was the institution with the largest number of publications, which might be an ideal candidate for academic collaboration. He Chuan, from the University of Chicago, had been influential in METTL3 research. Apart from cancers such as hepatocellular carcinoma, lipid diseases like obesity and atherosclerosis also showed high correlations with METTL3. In addition to m^6^A-related enzyme molecules, the key molecules with the highest frequency were C-MYC, EZH2, and PTEN, indicating that METTL3 is closely related to these molecules in human body. The abovementioned diseases and molecules were the frontiers of current research. METTL3 and METTL14 may function through opposite regulatory pathways in the same disease. “Leukemia,” “Liver Cancer,” and “Glioblastoma” may be potential research topics, and researchers should follow closely relevant studies to find more new findings in the coming years.
